# Checkmate to deliberate practice: the case of Magnus Carlsen

**DOI:** 10.3389/fpsyg.2014.00878

**Published:** 2014-08-14

**Authors:** Fernand Gobet, Morgan H. Ereku

**Affiliations:** ^1^Department of Psychological Sciences, University of LiverpoolLiverpool, UK; ^2^Department of Psychology, Brunel UniversityUxbridge, UK

**Keywords:** chess, deliberate practice, expertise, superior performance, talent

The role of practice in the acquisition of expertise has been a key research question at least since Bryan and Harter's (1899) study on expertise in Morse telegraphy, which proposed that it takes 10 years to become an expert. The framework of deliberate practice (Ericsson et al., 1993) has taken an extreme position by denying the role of talent in most domains and stating that superior performance is an increasing monotonic function of deliberate practice—the more goal-oriented practice, the higher the level of skill. For example, Ericsson et al. (1993) argue that “individual differences in ultimate performance can largely be accounted for by differential amounts of past and current levels of practice” (p. 392). The deliberate practice framework has captured the imagination of the popular press, as can be seen by the publication of several pop-science books such as *Talent is Overrated* (Colvin, 2008), *Outliers* (Gladwell, 2008) and *Bounce* (Syed, 2011).

In recent years, this framework has been criticized in academic circles; for example, in retrospective studies, the amount of deliberate practice accounts for only about one third of the variance in expertise in music and in chess (Hambrick et al., 2014). More naturalistic data also question the validity of the framework. As top performers have spent similar number of hours to improve and maintain their skills, the fact that individuals such as Roger Federer in tennis, Michael Jordan in basketball, Usain Bolt in sprint or Michael Schumacher in auto racing have so outrageously dominated their sport throws considerable doubt on the deliberate practice framework.

A particularly spectacular example is provided by chess grandmaster Magnus Carlsen (Norway), who became world champion in classic chess in November 2013 by beating Viswanathan Anand (India) and who also became world champion in rapid chess (15 min + 10 s additional time per move) and speed chess (3 min + 2 s additional time per move) in June 2014. In the June 2014 rating list published by the World Chess Federation (http://ratings.fide.com/toparc.phtml?cod=309), 23-year old Carlsen is ranked first with 2881 points^1^. This is just one point below 2882, the highest rating in chess history that Carlsen held in May 2014. There is a 66-point difference between him and the second player, grandmaster Levon Aronian (Armenia, 2815 points; see Table 1). This difference is nearly the same as that between the 2nd and the 14th player in the list (63 points), Dutch grandmaster Anish Giri (2752 points). Table 1 shows the rating of Carlsen and of the ten players following him in the list. A one-sample *t*-test confirms that Carlsen's rating is statistically different from the next ten grandmasters (*M* = 2780.6), *t*_(9)_ = −19.38, *p* < 0.001, *mean* difference = −100.4; 95% CI [−112.1, −88.7]. One hundred points is a considerable difference: it is half a standard deviation in skill and means that, against the very best players in the world, Carlsen's probability of winning is 63.7%.

**Table 1:** **Rank, country, rating, starting age, current age, and number of years of practice of the 11 top players in the world (June 2014)**.

**Rank**	**Name**	**Country**	**Rating**	**Starting age**	**Current age**	**Number of years of practice**
1	Carlsen, Magnus	Norway	2881	5	23	18
2	Aronian, Levon	Armenia	2815	9	31	22
3	Grischuk, Alexander	Russia	2792	4	30	26
4	Caruana, Fabiano	Italy	2791	5	21	16
5	Anand, Viswanathan	India	2785	6	44	38
6	Kramnik, Vladimir	Russia	2783	5	38	33
7	Nakamura, Hikaru	USA	2775	7	26	19
8	Topalov, Veselin	Bulgaria	2772	7	39	32
9	Karjakin, Sergey	Russia	2771	5	24	19
10	Vachier-Lagrave, Maxime	France	2762	4	23	19
11	Dominguez Perez, Leinier	Cuba	2760	8	30	22

*Source: http://ratings.fide.com/toparc.phtml?cod=309*.

To test the monotonic assumption, we collected information from the internet and biographies about the age at which these grandmasters started playing chess and about their current age (see Table 1). Starting age is a good approximation of when players started practicing seriously (i.e., using some form of deliberate practice), as most of these players obtained outstanding results in youth competitions a few years after starting playing chess, and indeed obtained the grandmaster title rapidly. In the case of Carlsen, he has stated that he had learned the rules at 5 years but started practicing seriously only at 8 years (see Gobet and Campitelli, 2007)^2^. To be consistent, we used starting age anyway. (Note that this bias adds years of deliberate practice, and thus is in a favor of the monotonic assumption.) If the monotonic assumption is correct, Carlsen should have accumulated more hours of deliberate practice than the other players, given the way he dominates the chess world. We did find that Carlsen's number of years of deliberate practice (18 years) is different to the average of the following ten best players in the world (*M* = 24.6 years), *t*_(9)_ = 2.83, *p* < 0.05, *mean* difference = 6.6 years; 95% CI [1.33, 11.87]. However, this result *is exactly the opposite of what is predicted by deliberate practice*: on average, Carlsen practiced statistically significantly *fewer years* than the other players. (Note also that, for the players in Table 1, the correlation between rating and the number of years of practice is negative (*r* = −0.21) but not statistically significant (*p* = 0.55)).

In this analysis, we have assumed that, at the top level, all players practice with extreme dedication and with the best training methods available. If expertise was solely a monotonic function of practice, then it follows that Carlsen, who learned the rules at age of five but started playing chess seriously at the relatively old age of eight, should be much weaker than most of the ten players that follow him in the international rating list, as these opponents had time to clock in substantially more deliberate practice (on average, at least 6.6 years more). The fact that Carlsen dominates the chess world so outrageously, being world champion not only in classic chess but also in rapid chess and in blitz, refutes this hypothesis, central to the theory of deliberate practice.

Several objections can be leveled at this analysis. We discuss three of them, and show that they do not invalidate our argument. First, Carlsen's prodigious skill throughout adolescence and early adulthood may not be as remarkable as it first appears, as numerous young players perform better that their older competitors. For example, Howard (1999) has shown that the top chess players are increasingly younger. Key changes have taken place in the last decades that enable more efficient practice (Gobet et al., 2002). In particular, the quality and quantity of chess books have dramatically increased over the last decades, and chess programs and computer databases have revolutionized training methods. That more efficient deliberate practice should lead to quicker progress is consistent with Ericsson et al.'s (1993) framework. However, as all players in the Table have benefitted from these improvements in training, this factor does not explain away Carlsen's superiority.

Second, it could be argued that, just like in sport, age plays an important role in chess and youth will give an edge to younger top competitors. It is known that the effects of ageing occur depressingly early with cognitive variables such as reasoning, visualization and processing speed, peak performance being observed in the early to mid-twenties (Salthouse, 2009). However, whether this is a key factor in chess is unclear, as six of the absolute top players shown in Table 1 are 30 years old or older. In addition, Gary Kasparov and Viswanathan Anand were still world champions when they were 37 and 44 years old, respectively. In any case, in Table 1 the correlation between age and rating (*r* = − 0.21) is not statistically significant (*p* = 0.54), but Carlsen is reliably younger than the other ten top players, *t*_(9)_ = 3.16, *p* < 0.05, *mean* difference = 7.6 years; 95% CI [2.16, 13.04]. Nevertheless, the age variable does not explain why Carlson is so clearly better than the four players who are roughly his age.

Third, Carlsen might have engaged in more intense deliberate practice. Although we do not know the details of Carlsen's training, this is unlikely, in particular if we use Ericsson et al.'s (1993) criterion that deliberate practice is not enjoyable. In a recent interview, Carlsen said that “in chess training, I do the things I enjoy. I don't particularly enjoy playing against computers, so I don't do that” (Anders, 2014). In addition, he is a keen sportsman, with a penchant for playing or watching football rather than practicing chess intensively (Sujatha, 2013).

Thus, the question arises, in the risk of offending the proponents of deliberate practice: Does Carlsen have a particular talent for chess? The answer to this question is so obvious in the chess world that it is not even posed—Carlsen is known as the “Mozart of chess.” Several factors support the hypothesis of talent. Carlsen showed clear signs of intellectual precocity early in his life. At the age of five, he knew “the area, population, flag, and capital of all the countries of the world,” and memorized similar information for all Norway's 430 municipalities (Agdestein, 2004, p. 10). He became a grandmaster just five years after starting playing chess seriously, at the age of 13 years and 148 days^3^. He has also adopted a highly unconventional approach to chess. While most grandmasters specialize in specific openings that they study at great length (Chassy and Gobet, 2011), often using computers, Carlsen plays a wide range of openings and avoids known variations, even accepting inferior positions as a consequence of this choice. Rather than preparing lengthy opening variations, he relies on his uncanny ability to find near-optimal moves in middle games and endgames. Together with scientific research, the case of Magnus Carlsen demonstrates that deliberate practice is necessary, but not sufficient, for achieving high levels of expert performance (Campitelli and Gobet, 2011).

## Conflict of interest statement

The authors declare that the research was conducted in the absence of any commercial or financial relationships that could be construed as a potential conflict of interest.

## Footnotes

^1^To measure chess players' skill level, the World Chess Federation (FIDE) uses the rating scale developed by Elo (1978), which is an interval scale that computes players' rating as a function of their results against other players of known rating. The scale has a normal distribution with a theoretical mean of 1500 and a standard deviation of 200 points. Grand Masters are typically rated above 2500 points. The best players of the world have around 2800 points and the weakest players less than 1200 points.

^2^Ericsson et al. (2007) explanation that prodigies' high levels of performance can be accounted for by the amount of deliberate practice made possible by a very early start does not apply in Carlsen's case.

^3^This contradicts another key prediction of the deliberate practice framework: “More specifically, expert performance is not reached with less than 10 years of deliberate practice” (Ericsson et al., 1993, p. 372).
